# Study of *Helicobacter pylori* genotype status in cows, sheep, goats and human beings

**DOI:** 10.1186/1471-230X-14-61

**Published:** 2014-04-03

**Authors:** Hassan Momtaz, Hossein Dabiri, Negar Souod, Mohsen Gholami

**Affiliations:** 1 Department of Microbiology, Faculty of Veterinary Medicine, ShahreKord Branch, Islamic Azad University, ShahreKord, Iran; 2 Department of Medical Microbiology, Faculty of Medicine, Shahid Beheshti University of Medical Science, Tehran, Iran; 3 Young Researchers and Elite club, Central Tehran Branch, Islamic Azad University, P.O.Box: 13185-768, Tehran, Iran; 4 Graduated of Veterinary Medicine, Faculty of Veterinary Medicine, ShahreKord Branch, Islamic Azad University, ShahreKord, Iran

**Keywords:** *Helicobacter pylori*, Virulence genes, Cow, Sheep, Goat, Human being

## Abstract

**Background:**

*Helicobacter pylori* is one of the most controversial bacteria in the world causing diverse gastrointestinal diseases. The transmission way of this bacterium still remains unknown. The possibility of zoonotic transmission of *H. pylori* has been suggested, but is not proven in nonprimate reservoirs. In the current survey, we investigate the presence of *H. pylori* in cow, sheep and goat stomach, determine the bacterium virulence factors and finally compare the human *H. pylori* virulence factors and animals in order to examine whether *H. pylori* might be transmitted from these animals to human beings.

**Methods:**

This cross- sectional study was performed on 800 gastric biopsy specimens of cows, sheep, goats and human beings. The PCR assays was performed to detection of *H. pylori*, *vacA* and *cagA* genes. The PCR products of Ruminant’s samples with positive *H. pylori* were subjected to DNA sequencing analysis. Statistical tests were applied for data analysis.

**Results:**

Overall 6 (3%) cows, 32 (16%) sheep and 164 (82%) human beings specimens were confirmed to be *H. pylori* positive; however we were not able to detect this bacterium in all 200 goat samples. The *vacA s1a/m1a* was the predominant *H. pylori* genotype in all three kinds of studied population. There was 3.4–8.4% variability and 92.9-98.5% homology between sheep and human samples.

**Conclusions:**

Considering the high sequence homology among DNA of *H. pylori* isolated from sheep and human, our data suggest that sheep may act as a reservoir for *H. pylori* and in the some extent share the ancestral host for the bacteria with human.

## Background

*Helicobacter pylori* is a gram negative, spiral shaped bacterium which its main reservoir is humans, particularly the human stomach. It colonizes most of the population, making it one of the most controversial bacteria in the world that cause gastritis, peptic ulcer, duodenal ulcer, lymphoma and gastric cancer [1].

According to the reports the main routs of infection has not been clarified yet [2]. However it is likely that *H. pylori* infection occurs during childhood or adolescence both in developing and developed countries [3] and its transmission occurs by person to person, either by fecal-oral or oral-oral routes [4].The possibility of zoonotic transmission of *H. pylori* has been suggested, but is not proven in nonprimate reservoirs [4]. Some reports indicated that there is a high prevalence of antibody against this bacterium in veterinarians, butchers and slaughters rather than other people, so it suggests that *H. pylori* might be transmitted from animals to human [5,6]. Recently some researchers have been isolated *H. pylori* from cow, sheep, camel, pigs and dogs milk [7,8]. Therefore, it seems that these animals can be a reservoir of this bacterium. The severity of clinical manifestations varies depends on several factors such as host genetic, immune system, bacterial load and virulence factors [9]. This bacterium has several virulence factor genes which are generally classified into three categories: I) strain-specific genes, such as *cag* pathogenesity island (PAI) and genes located in plasticity island region (e.g. *jhp0947* and *dupA* genes), which are present in only some *H. pylori* strains. II) phase-variable genes which change during different growth conditions. Based on comparison of three completed genomes of *H. pylori*, six genes encoding outer-membrane proteins (*babB*, *oipA*, *hopZ sabA*, *sabB* and *babC*) are supposed to have phase variation. III) The genes with polymorphisms, for instance, specific *vacA* genotypes have been associated with different clinical outcomes [10]. The *cag* pathogenicity island (PAI) which belongs to the first category, encodes a type IV secretion system [2,3]. The *cagA* gene is located in the end of the *cag* PAI and has been proposed as a marker for the *cag*PAI. Different type of the *cagA* gene in some region is associated with diverse clinical outcomes; for instance *cagA1a* in East Asian Strains, is associated with more severe clinical manifestations than the absence of the gene [11]. The other important virulence factor of *H. pylori* is a vacuolating cytotoxin (VacA), which belongs to the last category and is associated with injury to epithelial cells. The *vacA* gene is present in virtually all strains of *H. pylori* but it is polymorphic, comprising variable signal regions (type *s1* or *s2*) and mid-regions (type *m1* or *m2*). Type *s1/m1 vacA* contribute with more epithelial cell damage rather than type *s1/m2*, whereas type *s2/m2* and the rare *s2/m1* are supposed to be non-toxic due to the presence of a short 12-residue hydrophilic extension on the *s2* form [12,13]. The s-region is classified into *s1* and *s2* types and the m-region into *m1* and *m*2 types. The *s*1 type is further subtyped into *s1a*, *s1b* and *s1c* subtypes, and the *m1* into *m1a* and *m1b* subtypes. The mosaic combination of s and m-region allelic types determines the particular cytotoxin and, consequently, the pathogenicity of the bacterium [2,14].

In the current survey, we investigated the presence of *H. pylori* in cow, sheep and goat stomach, as well as bacterium virulence factors distribution among human and other studied population.

## Methods

### Population and sampling

Over all 800 samples; 200 from human and 600 from ruminant were included in the current study. In the ruminants group, over all 600 healthy domestic animals; 200 cows, 200 sheep and 200 goats referring for Zarrinshahr slaughterhouse in Isfahan, center of Iran, during February to August of 2012 were selected randomly. Considering sterile conditions, the sample from the rumen in size of 2 mm to 3 mm was obtained immediately after slaughtering. Samples were placed in 0.1 ml of sterile saline solution and were transported rapidly to the laboratory. The histological examinations were performed by the specialized veterinarians of Shahrekord Azad University. For analysis of *H. pylori* DNA from human origin, regardless to career of patients, two hundred patients with dyspepsia symptoms referring to gastroenterology department of Hajar Hospital Shahrekord, Iran, from December 2011 to April 2012 were selected and gastric biopsies from antrum were obtained during endoscopy by endoscopist. All patients provided written informed consent prior to endoscopy. All the specimens were placed in 0.1 ml of sterile saline solution and were transported to the laboratory immediately and were stored at -70°C until further investigation.

### DNA analysis

From all of biopsy specimens, DNA was extracted by using Genomic DNA purification kit (DNP™, CinnaGen, Iran) considering sterile condition according to manufacture recommendations. The *H. pylori* presence in studied samples was detected by PCR method using housekeeping gene; *glmM* gene as a target gene. Due to low sensitivity and difficulty of *H. pylori* culture particularly from animal sources, the samples were not cultured. The primers sequences for *glmM* gene amplification were as follows: GlmM-F (5′- GCTTACTTTCTAACACTAACGCGC-3′) and GlmM-R (5′- GGATAAGCTTTTAGGGGTGTTAGGGG-3′) [2]. Primers were used for PCR assays of *vacA* allels and *cag*A genes has been described before [15,16]. DNA samples *H. pylori* (D0008, Genekam, Germany) were used as positive control of *cagA* and *vacA* genes, and sterile distilled water was used as negative control. PCR was done in 20 μL (for *glmM*) or 25 μL (for *vacA* and *cagA*) of total reaction volume containing 1.5 mM MgCl_2_ (2.0 mM for *cagA*), 50 mM KCl, 10 mM Tris–HCl (pH 9.0), 0.1% Triton X-100, 200 μM dNTPs each (Fermentas), 0.4 μM primers, 0.3 U of Taq DNA polymerase (Fermentas), and 2 μL (40–260 ng/μL) of DNA. PCR was performed in a DNA Thermal Cycler (Eppendrof Mastercycler 5330, Eppendorf-Nethel-Hinz GmbH, Hamburg, Germany), with 40 cycles for GlmM primer and 35 cycles for *vacA* and *cagA* primers. Each cycle consisted of denaturation at 95°C/45 seconds; annealing at 59°C/30 seconds for *glmM*, 52°C/45 seconds for *vacA*, and 58°C/45 seconds for *cagA*; and extension at 72°C/45 seconds [16]. There was another longer extension of 6 minute at 72°C. PCR products were visualized by electrophoresis in 1% agarose gel, were stained with ethidium bromide, and were examined under ultraviolet illumination.

### DNA sequencing analysis

DNA sequencing analysis was performed on 6 *H. pylori* positive sample; 3 samples from cows and 3 samples from sheep which were selected randomly. Due to limitations we were not able to do sequence on all positive samples for *glmM* gene. For this purpose the DNA extraction was done by the same method as mentioned before for PCR. The amplified 296-bp PCR products (*glmM* gene) from 6 positive samples were purified with High pure PCR product purification kit (Roche Applied Science), according to manufacturer’s recommendations. Single DNA strands were sequenced with ABI 3730 XL device and Sanger sequencing method (Macrogen, Korea).

After the sequence of 6 isolates were trimmed by using Edit View v.1.0.1 (Applied Bioscience, Australia), the sequences of 8 isolate with human source, which has been stored in GenBank with accession numbers: FN598874, CP003476, DQ462665, M60398, NC017361, GU445163, DQ141576, AB664954 were aligned separately against obtained animal isolate sequences using the Clustal W v1.81 in order to obtain a consensus sequence for the gene, *glmM* (*H. pylori ureC*). BioEdit Pakage V.7.0.4.1 was used to edit all sequence alignments. The nucleotide sequences of the Iranian ruminant *H. pylori glmM* (*ureC*) gene was compared with the correspond sequences reported from other regions via NCBI. By using Njplot software and 1000 bootstrap replicate, an unrooted dendrogramme was constructed.

### Ethical considerations

The present study was accepted by the ethical committee of the Hajar Hospital of Shahrekord, Iran and Microbiology and Infectious Diseases Center of the Islamic Azad University of Shahrekord Branch, Iran. Written informed consent was obtained from all of the study patients or their parents.

## Results

Totally 600 ruminants and 200 human gastric samples were collected in the current investigation. According to clinical and histopathological examinations, 10 cows and 2 sheep had moderate gastric inflammations while all goats were healthy, however none of the animals showed clinical manifestations. Based on gastroendoscopic and histopathologic finding, out of 200 human biopsy specimens, sixteen patients (11.8%) had gastric ulcers, 22 (16.2%) had duodenal ulcers, 194 (97.5%) had chronic gastritis and 3 (2.2%) had gastric cancer. Among 200 cow samples and 200 sheep samples, 6 (3%) and 32 (16%) were confirmed to be *H. pylori* positive; however, we were not able to find any *H. pylori* in goat samples. Out of 200 humans samples, 164 (82%) were infected with this bacterium.

**Figure 1:**
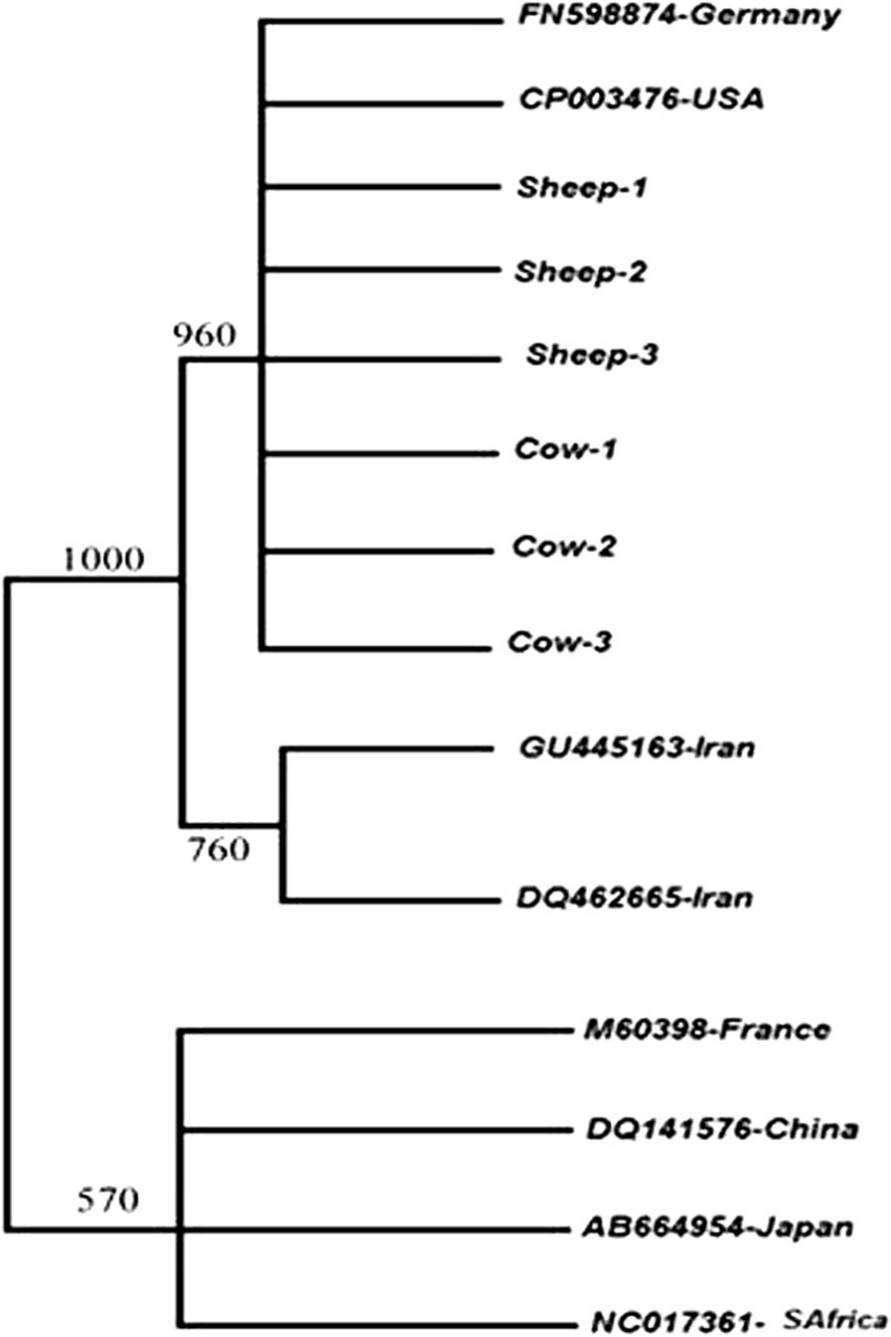
**Dendrogramme based on sequence alignment analysis of 6 Iranian ruminant strains and 8 of the human reference isolates from other regions of the world for *ureC *** **gene of *Helicobacterpylori*.**

When we came to analyze the *cagA* gene in the positive samples, out of 6 cow, 32 sheep and 164 human samples, positive for *H. pylori*, 4 (66.66%), 24 (75%) and 151 (92.08%) were *cagA*-positive respectively, however the *cagA* gene frequency among studied cow, sheep and human isolates was not statically significant (p = 0.7).In case of the *vacA* gene alleles, according to cow specimen results, the frequency of *vacA s1a*, *s1b*, *m1a* and *m2* were 5 (83.33%), 1 (16.66%), 2 (33.33%) and 4 (66.66%) respectively. We were not able to detect *vacA s1c*, *s2*, and *m1b* in the cows’ population. The frequency of *vacA s1a*, *s1b*, *s2*, *m1a* and *m2* were 16 (50%), 11 (34.37%), 5 (15.66%), 14 (43.75%) and 18 (56.25%) respectively in sheep’s population. The *s1c* and *m1b* did not amplify any band in PCR assay for sheep samples (Table 1). As it was indicated in Table 1, in isolates from human samples, 79 (48.17%) *s1a*, 21 (12.80%) *s1b*, 35 (21.34%) *s1c*, 29 (17.68%) *s2* were observed while for *vacA* m region, 52 (31.70%), 15 (9.14%) and 97 (59.14%) isolates showed *m1a*, *m1b* and *m2* genotype respectively. There was a statistically significant differences in prevalence of the *s1b* allele among human beings and cows isolates (*P* = 0.025) as well as *s1a/m2* genotypes among human beings and sheep strains (*P* = 0.04). There was no statically significant relation between genotypes of *H. pylori* recovered from cows compare to sheep (*P* = 0.81) (Table 2). The nucleotide sequences of *H. pylori glmM* gene, obtained from 6 Iranian ruminants; 3 cows and 3 sheep were compared with those from the known human reference sequences obtained from the GenBank nucleotide sequence database (8 sequences corresponding to *H. pylori*). The nucleotide sequence analyses showed a variability of 0.7–1.4% for the *ureC* gene between sheep and cows samples (Table 3) and variations was consisted only in nucleotide sub-situation. Frame shift, deletion, insertion and nonsense mutations were not observed. When we compared the sequences of the *ureC* gene in sheep and human *H. pylori* isolates; there was 3.4–8.4% variability and 92.9-98.5% homology. The greatest sequence similarity (98.5%,) was found between *H. pylori* isolates of Iranian sheep and German human (FN598874), while the lowest relationship (91.6%) between Iranian cow *ureC* sequence and South Africa (NC017361) was observed (Figure 1).

**Table 1:** **The frequency of *cagA *** **and *vacA *** **alleles in *Helicobacter pylori*** **of ruminants and human samples**

**Positive samples**	***cagA***	***s1a***	***s1b***	***s1c***	***s2***	***m1a***	***m1b***	***m2***
**Cow**	4	5	1	0	0	2	0	4
**6 (3%)**	(66.66%)	(83.33%)	(16.66%)			(33.33%)		(66.66%)
**Sheep**	24	16	11	0	5	14	0	18
**32 (16%)**	(75%)	(50%)	(34.37%)		(15.62%)	(43.75%)		(56.25%)
**Human**	151	79	21	35	29	52	15	97
**164 (82%)**	(92.08%)	(48.17%)	(12.80%)	(21.34%)	(17.68%)	(31.70%)	(9.14%)	(59.14%)

**Table 2:** **The frequency of *vacA *** **genotypes in *Helicobacter pylori *** **of ruminants and human samples**

**Positive samples**	***s1a/m1a***	***s1a/m1b***	***s1a/m2***	***s1b/m1a***	***s1b/m1b***	***s1b/m2***	***s1c/m1a***	***s1c/m1b***	***s1c/m2***	***s2/m1a***	***s2/m1b***	***s2/m2***
**Cow**	2	0	3	0	0	1	0	0	0	0	0	0
**6 (3%)**	(33.33%)		(50%)			(16.66%)						
**Sheep**	6	0	10	5	0	6	0	0	0	1	0	4
**32 (16%)**	(18.75%)		(31.25%)	(15.62%)		(18.75%)				(3.12%)		(12.5%)
**Human**	27	8	45	7	5	10	12	4	18	6	0	22
**164 (82%)**	(16.46%)	(4.87%)	(27.43%)	(4.26%)	(3.04%)	(6.09%)	(7.31%)	(2.43%)	(10.97%)	(3.65%)		(13.41%)

**Table 3:** **Sequence identity matrix of partial *ureC *** **gene of Iranian ruminant *Helicobacter pylori *** **in comparison with 8 known human reference sequences**

**Seq**	**Cow-1**	**Cow-2**	**Cow-3**	**Sheep-1**	**Sheep-2**	**Sheep-3**	**FN598874-Germany**	**CP003476-USA**	**DQ462665-Iran**	**GU445163-Iran**	**M60398-France**	**NC017361-S Africa**	**DQ141576-China**	**AB664954-Japan**
**Cow-1**	ID	0.999	1	0.989	0.987	0.991	0.953	0.962	0.932	0.941	0.928	0.920	0.926	0.926
**Cow-2**	0.999	ID	0.999	0.987	0.988	0.986	0.950	0.966	0.936	0.940	0.929	0.918	0.924	0.923
**Cow-3**	1	0.999	ID	0.992	0.986	0.993	0.927	0.964	0.932	0.944	0.930	0.916	0.922	0.923
**Sheep-1**	0.989	0.987	0.991	ID	1	0.998	0.983	0.979	0.976	0.973	0.967	0.937	0.952	0.954
**Sheep-2**	0.987	0.988	0.986	1	ID	0.999	0.985	0.981	0.978	0.981	0.966	0.934	0.958	0.959
**Sheep-3**	0.991	0.986	0.993	0.998	0.999	ID	0.980	0.983	0.969	0.980	0.964	0.929	0.961	0.962
**FN598874-Germany**	0.953	0.950	0.957	0.983	0.985	0.980	ID	0.986	0.992	0.991	0.985	0.983	0.976	0.984
**CP003476-USA**	0.962	0.966	0.964	0.979	0.981	0.983	0.986	ID	0.990	0.993	0.984	0.979	0.980	0.983
**DQ462665-Iran**	0.932	0.936	0.932	0.976	0.978	0.969	0.992	0.990	ID	1	0.982	0.969	0.983	0.986
**GU445163-Iran**	0.941	0.940	0.944	0.973	0.981	0.980	0.991	0.993	1	ID	0.985	0.970	0.986	0.989
**M60398-France**	0.928	0.929	0.930	0.967	0.966	0.964	0.985	0.984	0.982	0.985	ID	0.975	0.990	0.991
**NC017361-S Africa**	0.920	0.918	0.916	0.937	0.934	0.929	0.983	0.979	0.969	0.970	0.975	ID	0.993	0.989
**DQ141576-China**	0.926	0.924	0.922	0.952	0.958	0.961	0.976	0.980	0.983	0.986	0.990	0.993	ID	0.994
**AB664954-Japan**	0.926	0.923	0.923	0.954	0.959	0.962	0.984	0.983	0.986	0.989	0.991	0.989	0.994	ID

## Discussion

Human is known as the main host of *H. pylori*; however the histopathology of this bacterium is in contrasts with many other gastric *Helicobacter* spp. In which their natural host response against bacteria involve mild or even no inflammatory response [4], so it is possible that *H. pylori* has originated from another mammalian host in the distant past [17]. In the current survey we evaluated whether cow, sheep and goat can be the original host of this bacterium. In order to achieve this goal we collected 600 gastric specimens from healthy cows, sheep, goats and 200 gastric biopsy from human with dyspepsia in west of Iran where the prevalence of *H. pylori* is higher than 70% [18]. In our investigation, the prevalence of *H. pylori* in cows and sheep’s population was 3% and 16% respectively, we were not able to detect it in goat’s gastric tissue samples whereas the Italian survey on 400 milk samples by nested PCR assay, indicated that the prevalence of this bacterium in cow, sheep and goat populations were 50%, 33% and 25.6% respectively [8]. Scandinavian researchers found *H. pylori* in 60% of 38 sheep gastric tissue [4]. Also Rahimi and Kheirabadi declared *H. pylori* existed in 12.2% of sheep, 8.7% of goat and 14.1% of cow milk by PCR method [19]. In the other study which was conducted in Japan, *H. pylori* was found in 72.2% of cow raw milk specimens [20]. The diversity of *H. pylori* frequency in various hosts and regions may relate to animal, microbe and environmental factors. The prevalence of this bacterium in Iranian patients was 82% which is similar to previous reports from Iran and also Japan, South America, Turkey and Pakistan where more than 80% of dyspepsia patients were *H. pylori* positive, however in Scandinavia and England the prevalence ranges between 20% to 40% [21,22]. According to our results, screening of goats’ stomach for *H. pylori* was negative which is in accordance with Gueneau et al. study in 2003 in which they failed to detect *H.pylori* in studied goats [23]. This finding may support by two possible reasons: Ones that goats are an exception among ruminants in having particular natural mechanisms of resistance to this bacterium. Another hypothesis is that some other microorganisms like *Candidatus H. bovis* may colonize the goat’s stomach and establish the extent of the resistance of goats to the super infection with *H. pylori *[23]. *H. pylori* strains with the *cagA* gene is supposed to be more virulent rather than *cagA*-negative strains [12] however this is not constant [2]. The prevalence of *cagA*-positive *H. pylori* varies from one geographic region to another, e.g., 97% in Korea, 94% in Malaysia, 90% in China, 78% in Turkey, 53% in Kuwait, 85% North America and 65% in Slovenia [14,24-27]. In the current study *cagA* gene was found in 92% of Iranian populations which is in accordance with previous local report [18]. Since the most of *H. pylori* isolated from human samples regardless to clinical outcomes harbor the *cagA* gene (*P* > 0.05), thereby as it was declared previously, our finding did not support the role of the *cagA* as predictive marker for increased virulence feature of *H. pylori* in Iranian dyspepsia patients [1]. The *cagA* gene was found in 66%, 75% of cow and sheep populations respectively, which was not studied on animals’ samples yet. There was no statically meaningful difference in status of the *cagA* in human and animal samples, which may reflect that all *H. pylori* recovered from human and animals have same ancestors. According to our results for the *vacA*, all of our samples with positive PCR for *H. pylori*, irrespective to source of strains was positive for *vacA*. Although Dore et al. [4] in 2001 detected *vacA* gene in 60.3% and 7.9% of *H. pylori* strains isolated from sheep tissue and sheep milk samples respectively; now it is supposed that all *H. pylori* strains should possess the *vacA* gene, as it was supported by many studies around the world [12,26,27]. The *vacA s1a/m2* were predominant *vacA* alles among all three studied population including human, cow and sheep.

Based on statistical analyses, there was a significant correlation between *s1a/m2* genotype of *H. pylori* in sheep and human beings. Also *s1a* allele was significantly prevalent among cow and human. To our knowledge, this is the first comparison study of *H pylori* DNA sequence among specimens from cow, sheep and human in Middle East. As it was shown in Table 3 there was a high DNA sequence homology between *H. pylori* strains of sheep and human however this homology was low between cows and humans. The rate of homology was low between cow and sheep too. Since considerable number of studied sheep carried *H. pylori* without any pathological evidence, it seems that sheep may are natural host for *H. pylori*. Besides DNA sequence homology among sheep and human *H. pylori* strains suggest that sheep may serve as a reservoir for this bacteria. Our findings are consistent with Dore et al. study which has hypothesized sheep is the ancestor host of *H. pylori *[4]. Although high prevalence of *H. pylori* among human population in comparison with other mammalian, indicating *H. pylori* is more adapted to human body, the main role of sheep in *H. pylori* evolution story is supported by our study in company with some other studies. Dore et al. showed that nearly all of Sardinian shepherds carried *H. pylori*. Morris et al. has reported the higher prevalence of antibodies against *H. pylori* in abattoir workers, such as veterinarians, butchers, and slaughterers [28]. Mégraud and Broutet study showed a number of animals, mostly living in human environment, had H*. pylori* in their stomach and therefore to be involved in the transmission of this bacterium [29]. Some other reports also support zoonotic transmission of *H. pylori via* close contact with domestic animals [4,30-33]. These studies, along with those have been shown that *H. pylori* can survive in sheep milk [4,19,34-36] are supportive for reservoir role of sheep for human infection. Due to lack of any recorded sequence for *H. pylori* with animal source in Gene bank, we compared our isolates sequences with recorded human isolates sequences. Despite the little diversity in studied sequences, we were able to justify the genetic diversity of the bacterium based on its diverse hosts. As the origin of many Iranian noble cows and sheep refer to the America and Germany, so the perceived genetic similarities among sequences of *H. pylori* FN598874-Germany and CP003476-USA with those of Iranian cow and sheep isolates in this research can justify this claim. On the other hand, transportation of livestock between Far-East Countries (Japan and China) and South Africa and Iran basically does not have historical background [37]. Thus, placing of Japanese, Chinese and South Africans strains in other branches of phylogenetic tree is indicating more differences in the sequence of *H. pylori* between Iran and mentioned countries.

## Conclusion

In conclusion cows and sheep in Iran harbor *H. pylori* in their gastric tissue similar in genotype of the *cagA* and *vacA* allels with isolates recovered from human. Also since there was a high homology sequence of *H. pylori* DNA among sheep and human, suggest that sheep may are the natural reservoir of the bacteria and can transmit *H. pylori* to human community.

## Competing interests

The authors declare that they have no competing interests.

## Authors’ contributions

The DNA extraction, PCR techniques and supporting of project were performed by HM and HD. MG collected the Samples, Statistical analysis and writing of manuscript were performed by NS, All authors read and approved the final manuscript.

## Pre-publication history

The pre-publication history for this paper can be accessed here:

http://www.biomedcentral.com/1471-230X/14/61/prepub
